# Inefficient HIV-1 *trans* Infection of CD4^+^ T Cells by Macrophages from HIV-1 Nonprogressors Is Associated with Altered Membrane Cholesterol and DC-SIGN

**DOI:** 10.1128/JVI.00092-18

**Published:** 2018-06-13

**Authors:** Diana C. DeLucia, Charles R. Rinaldo, Giovanna Rappocciolo

**Affiliations:** a Department of Infectious Diseases and Microbiology, Graduate School of Public Health, University of Pittsburgh, Pittsburgh, Pennsylvania, USA; Emory University

**Keywords:** cholesterol, DC-SIGN, HIV-1, simvastatin, disease progression, free cholesterol, macrophage, nonprogressors, *trans* infection

## Abstract

Professional antigen-presenting cells (APC; myeloid dendritic cells [DC] and macrophages [MΦ]; B lymphocytes) mediate highly efficient HIV-1 infection of CD4^+^ T cells, termed *trans* infection, that could contribute to HIV-1 pathogenesis. We have previously shown that lower cholesterol content in DC and B lymphocytes is associated with a lack of HIV-1 *trans* infection in HIV-1-infected nonprogressors (NP). Here, we assessed whether HIV-1 *trans* infection mediated by another major APC, MΦ, is deficient in NP due to altered cholesterol metabolism. When comparing healthy HIV-1 seronegatives (SN), rapid progressors (PR), and NP, we found that monocyte-derived MΦ from NP did not mediate HIV-1 *trans* infection of autologous CD4^+^ T cells, in contrast to efficient *trans* infection mediated by SN and PR MΦ. MΦ *trans* infection efficiency was directly associated with the number of DC-specific intercellular adhesion molecule-3-grabbing nonintegrin (DC-SIGN)-expressing MΦ. Significantly fewer NP MΦ expressed DC-SIGN. Unesterified (free) cholesterol in MΦ cell membranes and lipid rafting was significantly lower in NP than PR, as was virus internalization in early endosomes. Furthermore, simvastatin (SIMV) decreased the subpopulation of DC-SIGN^+^ MΦ as well as *cis* and *trans* infection. Notably, SIMV decreased cell membrane cholesterol and led to lipid raft dissociation, effectively mimicking the incompetent APC *trans* infection environment characteristic of NP. Our data support that DC-SIGN and membrane cholesterol are central to MΦ *trans* infection, and a lack of these limits HIV-1 disease progression. Targeting the ability of MΦ to drive HIV-1 dissemination in *trans* could enhance HIV-1 therapeutic strategies.

**IMPORTANCE** Despite the success of combination antiretroviral therapy, neither a vaccine nor a cure for HIV infection has been developed, demonstrating a need for novel prophylactic and therapeutic strategies. Here, we show that efficiency of MΦ-mediated HIV *trans* infection of CD4^+^ T cells is a unique characteristic associated with control of disease progression, and it is impaired in HIV-infected NP. *In vitro* treatment of MΦ from healthy donors with SIMV lowers their cholesterol content, which results in a strongly reduced *trans* infection ability, similar to the levels of MΦ from NP. Taken together, our data support the hypothesis that MΦ-mediated HIV-1 *trans* infection plays a role in HIV infection and disease progression and demonstrate that the use of SIMV to decrease this mechanism of virus transfer should be considered for future HIV therapeutic development.

## INTRODUCTION

The development and implementation of combination antiretroviral therapy (ART), which can effectively lower HIV-1 viral load to undetectable levels, has greatly decreased the morbidity and mortality associated with HIV-1 infection. Even with ART-mediated viral suppression, however, there is a reservoir of HIV-1-infected CD4^+^ T lymphocytes that contributes to incomplete viral clearance or eradication (1–5). Without ART, fewer than 5% of infected individuals can control HIV-1 infection and greatly slow or prevent progression to AIDS (6). Collectively referred to as nonprogressors (NP), this is a heterogeneous group characterized by having either consistently undetectable levels of HIV-1 RNA (elite controllers), 50 to 2,000 plasma HIV-1 RNA copies/ml (viremic controllers), or CD4^+^ T cell counts of >500/mm^3^ (long-term nonprogressors).

HIV-1 T cell-to-T cell *trans* infection is believed to be a critical factor contributing to viral persistence during ART (7, 8). However, *in vitro* CD4^+^ T cell *trans* infection mediated by professional antigen-presenting cells (APC), i.e., dendritic cells (DC), macrophages (MΦ), and B lymphocytes, results in much higher virus replication in T cells than in either T cell-to-T cell *trans* infection or direct *cis* infection of T cells (9). It is plausible that such transfer of virus during direct cell-to-cell contact through the infectious synapse represents a mechanism to evade immune responses, particularly in lymphoid tissue, thereby aiding the maintenance of an infected CD4^+^ T cell latent HIV-1 reservoir. Thus, elucidation of *trans* infection mechanisms could provide novel targets for prophylactic and therapeutic medicine, as well as reveal potential methods for identifying and eliminating the viral reservoir.

Cellular cholesterol is essential for HIV-1 *trans* infection of CD4^+^ T cells mediated by DC and B cells (10). Research has focused on the impact of cholesterol content in virion envelopes on HIV-1 infection and pathogenesis (11, 12) and characterized the association of cholesterol with binding, entry, and budding of HIV-1 particles from target CD4^+^ T cells. Although elegant studies have demonstrated that MΦ mediate highly efficient HIV-1 *trans* infection (13–15), there is no information on the role of cholesterol in this process. We have demonstrated that DC and B cells of NP do not *trans* infect autologous or heterologous CD4^+^ T cells. We discovered a unique association of decreased DC and B cell total cholesterol content and their inability to *trans* infect (10). While past research has focused on the impact of virion envelope cholesterol content on HIV-1 infection and pathogenesis (11, 12), there is no information on MΦ *trans* infection and cholesterol content related to HIV-1 disease progression. Here, we demonstrate that MΦ are similar to the other APC in the inability to *trans* infect T cells in NP. This deficiency is cholesterol dependent as well as being related to low expression of DC-specific intercellular adhesion molecule-3-grabbing nonintegrin (DC-SIGN), a C-type lectin that serves as a receptor for HIV-1 on APC (16).

## RESULTS

### MΦ-mediated HIV-1 *trans* infection enhances virus production from CD4^+^ T cells in SN.

**FIG 1:**
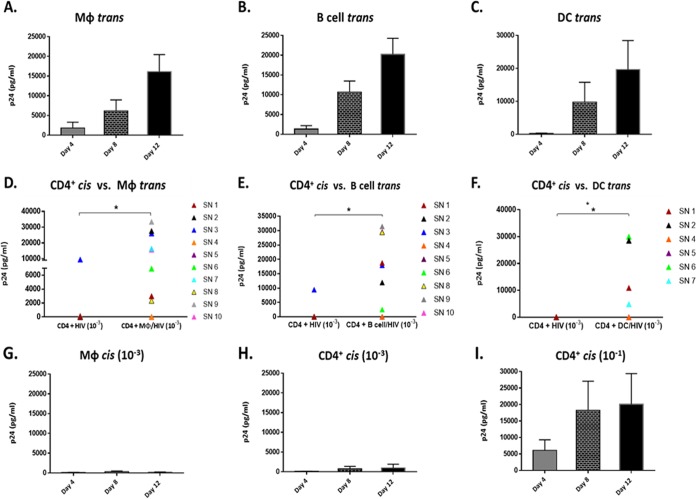
MΦ-mediated *trans* infection enhances virus production in autologous CD4^+^ T cells in SN. (A to C) HIV-1 *trans* infection in MΦ (*n* = 8 SN) (A), B cells (*n* = 7 SN) (B), and DC (*n* = 6 SN) (C) loaded with HIV-1 BaL (MOI, 10^−3^) and cocultured with activated, autologous CD4^+^ T cells for 12 days. Supernatants were assessed for p24 concentration at days 4, 8, and 12. (D to F) CD4^+^ T cells loaded with the low concentration of HIV-1 BaL (MOI, 10^−3^) and analyzed for *cis* infection compared to *trans* infection mediated by MΦ (D), B cells (E), and DC (F) at day 12. (G) MΦ loaded with HIV-1 BaL (MOI, 10^−3^) and assessed for *cis* infection (*n* = 10). (H) CD4^+^ T cells were loaded with HIV-1 BaL (MOI, 10^−3^) and assessed for *cis* infection (*n* = 10). (I) CD4^+^ T cells loaded with a higher concentration of HIV-1 BaL (MOI, 10^−1^) as a *cis* control to assess susceptibility to infection (*n* = 10). *, *P* < 0.05. Histograms represent the means ± standard errors (SE).

To establish our model for assessing HIV-1 *trans* infection mediated by MΦ, we cultured monocyte-derived MΦ from 10 SN recruited from the Multicenter AIDS Cohort Study (MACS) (Table 1). MΦ were loaded with a low multiplicity of infection (MOI) of HIV-1 R5-tropic BaL (MOI, 10^−3^), followed by coculture with autologous CD4^+^ T cells and assessment of HIV-1 p24 core antigen in cell-free supernatant. We chose to load MΦ with an MOI of 10^−3^ because it is suboptimal for efficient *cis* infection of CD4^+^ T cells yet is highly effective in APC-T cell *trans* infection (10). Under these conditions, MΦ-mediated *trans* infection of HIV-1 to autologous CD4^+^ T cells was detected in eight of 10 SN. The *trans* infection of the eight SN was demonstrated by detectable HIV-1 p24 by 4 to 12 days of coculture (Fig. 1A). This was comparable to our established models of *trans* infection mediated by activated B cells (Fig. 1B) and monocyte-derived DC (Fig. 1C) from the same SN. By day 12 of coculture, MΦ-mediated *trans* infection of CD4^+^ T cells was significantly greater (*P* < 0.05) than virus production in *cis*-infected CD4^+^ T cells (Fig. 1D). Moreover, as seen with MΦ, both B cell- and DC-mediated *trans* infection enhanced overall production of p24 compared to T cell *cis* infection (Fig. 1E and F).

**TABLE 1:** Virological and genotypic characterization of SN, NP, and PR

Participant	Infection duration^*a*^ (yr)	CD4^+^ T cell count (mean ± SE)	HIV-1 load (mean ± SE)	CCR5Δ32 genotype	HLA B locus genotype
SN1	NA	1,200 ± 206	NA	WT/WT	0801/5501
SN2	NA	987 ± 142	NA	WT/WT	0702/1501
SN3	NA	770 ± 64	NA	WT/WT	3503/4403
SN4	NA	924 ± 94	NA	WT/WT	2705/4403
SN5	NA	615 ± 40	NA	WT/WT	3801/3801
SN6	NA	1,198 ± 168	NA	WT/WT	1402/5802
SN7	NA	786 ± 101	NA	WT/WT	4001/5501
SN8	NA	683 ± 95	NA	WT/WT	5001/5201
SN9	NA	924 ± 233	NA	WT/WT	3501/5501
SN10	NA	928 ± 71	NA	WT/WT	2705/4501
NP1	>8	924 ± 62	3,055 ± 566	WT/WT	3910/5301
NP2	>8	743 ± 59	3,242 ± 1,931	WT/WT	3501/8101
NP3	>29	1,259 ± 62	4,764 ± 4,432	WT/WT	1402/4701
NP4	>8	1,007 ± 31	571 ± 90	WT/WT	1501/4201
NP5	>18	759 ± 57	20,893 ± 7,585	Δ32/WT	1501/4402
PR1	7	232 ± 49	396,572 ± 136,552	WT/WT	5001/5701
PR2	>13	415 ± 119	12,507 ± 8019	WT/WT	1401/5101
PR3	>6	335 ± 154	13,524 ± 9606	WT/WT	0801/0801
PR4	>7	424 ± 135	25,643 ± 3,935	WT/WT	4403/5601
PR5	4	598 ± 387	188,576 ± 119,553	WT/WT	0801/3503

a Years HIV-1 seropositive prior to ART while enrolled in the Multicenter AIDS Cohort Study (MACS). NA, not applicable; >, individuals were HIV seropositive upon enrollment into the MACS. Mean CD4^+^ T cell counts and mean HIV-1 loads were calculated from compiled data from all visits while enrolled in the MACS prior to ART.

Notably, none of the three types of APC mediated detectable *trans* infection in 2 of the 10 SN tested (SN4 [orange] and SN5 [purple]) (Fig. 1D, E, and F). These results were confirmed by repeated testing of APC and T cells from these same MACS participants obtained at different clinic visits.

MΦ loaded with the same low concentration of HIV-1 (MOI, 10^−3^) and cultured alone for *cis* infection in parallel to each *trans* infection experiment demonstrated negative or minimal levels of p24 for all SN (Fig. 1G). We also did not detect infection in *cis*-exposed CD4^+^ T cells at the low MOI (10^−3^) (Fig. 1H), with the exception of those for one participant (SN3). As expected, CD4^+^ T cells from all SN, including SN4 and SN5, were susceptible to *cis* infection with a high concentration of HIV-1 (MOI, 10^−1^) (Fig. 1I). Taken together, these data support that both myeloid- and lymphoid-derived APC share a feature essential for HIV-1 *trans* infection independent of susceptibility of T cells to *cis* infection. They also indicate that APC from some SN have an inherent inability to *trans* infect T cells.

### MΦ mediate HIV-1 *trans* infection in PR but not NP.

Previous work from our laboratory demonstrated that DC- and B cell-mediated *trans* infection was defective in NP while being completely functional in PR (10). Therefore, we sought to determine if MΦ-mediated *trans* infection was also altered in NP compared to that in PR and SN. We assessed *trans* infection efficiency of MΦ from 5 PR, 5 NP, and 10 SN (Table 1). Peripheral blood mononuclear cell (PBMC) samples were acquired while individuals were ART naive and not taking cholesterol-lowering medication.

**FIG 2:**
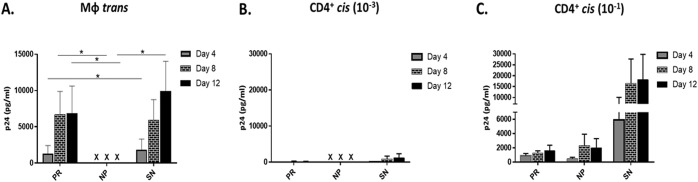
MΦ-mediated *trans* infection is negative in NP compared to PR and SN. (A) MΦ from PR, NP, and SN were loaded with HIV-1 BaL (MOI, 10^−3^) and cocultured with activated autologous CD4^+^ T cells for 12 days. Supernatant was assessed for p24 concentrations at days 4, 8, and 12. (B) CD4^+^ T cells were loaded with HIV-1 BaL (MOI, 10^−3^) in *cis* as a control for coculture *trans* infections. (C) CD4^+^ T cells were loaded in *cis* with a higher concentration of HIV-1 BaL (MOI, 10^−1^) to assess susceptibility to infection. PR, *n* = 5; NP, *n* = 5; SN, *n* = 10. *, *P* ≤ 0.05. **X**, below the limit of detection. Histograms represent the means ± SE.

MΦ exposed to the low concentration (MOI 10^−3^) of HIV-1 were cocultured with autologous CD4^+^ T cells for 12 days; coculture supernatants were assessed over time for HIV-1 p24 production. Under these conditions, MΦ-mediated *trans* infection was undetectable in all NP tested throughout the 12 days of coculture, while MΦ from PR were able to *trans* infect autologous CD4^+^ T cells. In addition, MΦ *trans* infection in PR was not significantly different from that of SN beyond day 4 (Fig. 2A). Levels of *cis* infection controls of CD4^+^ T cells alone with the low MOI used for *trans* infection experiments were very low or undetectable in all clinical groups (Fig. 2B). However, CD4^+^ T cells from all PR and NP (Fig. 2C) were susceptible to *cis* infection at a higher MOI (10^−1^). Taken together, these data show that MΦ derived from NP do not efficiently transfer HIV-1 to autologous CD4^+^ T cells, which are capable of supporting HIV-1 CD4^+^ T *cis* infection, in accordance with our previous DC and B cell results (10).

### MΦ susceptibility to HIV-1 *cis* infection is associated with HIV-1 disease progression.

**FIG 3:**
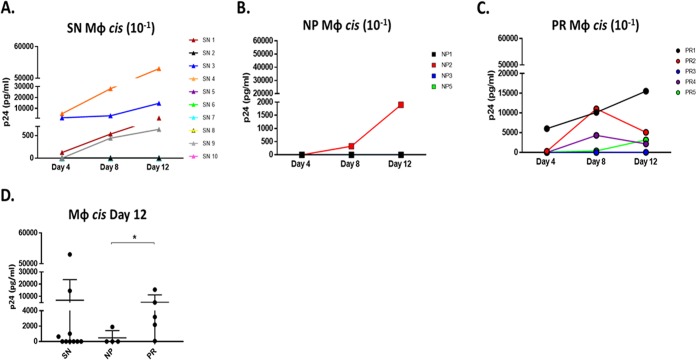
MΦ *cis* infection is significantly higher in PR than NP. (A to C) SN MΦ (A), NP MΦ (B), and PR MΦ (C) were loaded with the higher concentration of HIV-1 BaL (MOI, 10^−1^) and cultured, and supernatants were analyzed by p24 ELISA at days 4, 8, and 12. (D) p24 content was compared in day 12 supernatant of SN (*n* = 10), NP (*n* = 4), and PR (*n* = 5). *, *P* ≤ 0.05. Histograms represent the means ± SE.

MΦ susceptibility to HIV-1 *cis* infection was assessed across the three study groups. MΦ were exposed to HIV-1 BaL (MOI, 10^−1^) and cultured for 12 days. Only 4 of the 10 SN exhibited MΦ *cis* infection (Fig. 3A). Of the 6 individuals with no detectable *cis* infection, 5 had efficient *trans* infection. Additionally, no correlation was found between MΦ *trans* and *cis* infection efficiency in SN (data not shown), suggesting the susceptibility of MΦ to HIV-1 *cis* infection does not determine *trans* infection ability. MΦ *cis* infection was only detected in 1 NP (NP2) (Fig. 3B). However, it was detected in 4 of 5 PR (PR1, PR2, PR4, and PR5) (Fig. 3C). Importantly, compared to results on day 12, the magnitude of MΦ-positive *cis* infection was significantly higher in PR than NP (Fig. 3D). These findings further support a novel divergence of MΦ function among PR and NP and its correlation with disease progression.

### The number of DC-SIGN-expressing MΦ correlates with *trans* infection efficiency.

**FIG 4:**
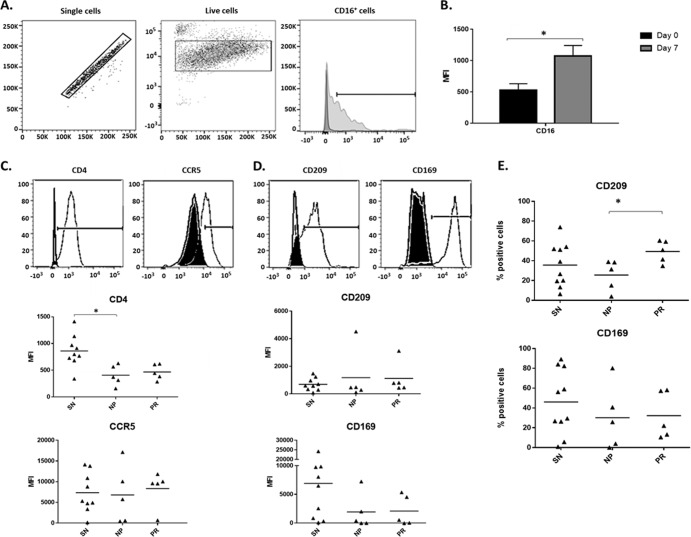
Number of DC-SIGN^+^ MΦ is lower in NP than in PR. (A) Gating strategy and live cell selection for MΦ phenotype by flow cytometry. (B) CD16 surface expression level (MFI) of SN CD14^+^ monocytes (day 0) and MΦ (day 7) was assessed by flow cytometry (*n* = 9). (C and D) Representative fluorescence intensity histograms (isotype, black; experimental, white) and individual MFI of MΦ measured by flow cytometry at day 7 for surface expression of CD4 and CCR5 (C) and CD209 (DC-SIGN) and CD169 (Siglec-1) (D). (E) Percentage of MΦ displaying surface expression of DC-SIGN and Siglec-1 was analyzed by flow cytometry and compared among SN (*n* = 9 or 10), NP (*n* = 5), and PR (*n* = 5). *, *P* ≤ 0.05. Histograms represent the means ± SE.

To identify potential factors influencing MΦ *trans* and *cis* infection efficiency, MΦ were phenotyped by flow cytometry. To assess MΦ differentiation, CD16 expression, which is low on monocytes and high on macrophages (17), was compared on CD14^+^ monocytes (day 0) and cultured macrophages (day 7). A significant increase in CD16 expression levels (mean fluorescence intensity, or MFI) was observed in the cultured macrophages (Fig. 4A and B). *trans* infection of CD4^+^ T cells is believed to occur by two different, but not mutually exclusive, pathways following DC or MΦ exposure to virus: (i) exposure through rapid virus uptake via endocytosis into vesicles and (ii) after *de novo* infection (9). Because MΦ are both susceptible to *cis* infection with R5-topic HIV-1 and capable of *trans* infection, cells were analyzed for classic markers associated with *cis* infection as well as HIV-1 endocytosis. MΦ surface expression of the primary HIV-1 receptor CD4, and coreceptor CCR5 for R5-tropic HIV BaL, was analyzed. CD4 expression level was significantly lower in HIV-1-infected NP than SN, and was similar to MΦ expression in HIV-1-infected PR, while CCR5 expression was similar among the three groups (Fig. 4C).

The C-type lectin DC-SIGN facilitates endocytosis-mediated HIV-1 *trans* infection (18, 19). The sialic acid binding adhesion molecule Siglec-1 has also been implicated in HIV-1 *trans* infection (20). Therefore, we assessed surface expression of both DC-SIGN and Siglec-1 on MΦ. Levels of the glycoproteins did not differ among the three groups (Fig. 4D). However, a significantly lower percentage of NP than PR MΦ were DC-SIGN positive, while no difference was seen in the percentage of Siglec-1-positive cells (Fig. 4E). MFI of all four markers was variable within SN (Fig. 4C and D) but did not correlate with MΦ *trans* infection efficiency (data not shown). Collectively, these data suggest that the lack of MΦ-mediated *trans* infection seen in NP is associated with the number of MΦ expressing DC-SIGN, a protein known to be involved in HIV-1 attachment (18, 21) and endocytosis (22).

### MΦ *trans* and *cis* infections of HIV-1 are facilitated by DC-SIGN.

**FIG 5:**
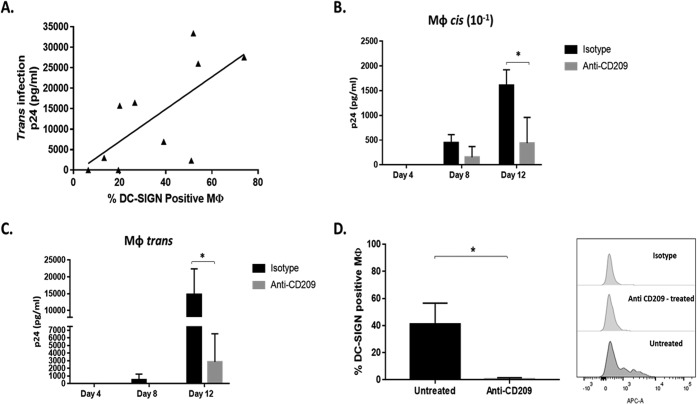
Number of DC-SIGN^+^ MΦ is positively correlated with MΦ-mediated *cis* and *trans* infection efficiency. (A) SN MΦ-mediated *trans* infection efficiency and percentage of DC-SIGN-positive MΦ were assessed for association by linear regression (*P* = 0.03, *F* = 7.18, *R*^2^ = 0.47) (*n* = 10). (B and C) Anti-DC-SIGN- and isotype control-treated SN MΦ were loaded with HIV-1 BaL (MOI, 10^−1^) and cultured alone, or loaded at an MOI of 10^−3^ and cultured with activated autologous CD4^+^ T cells, to assess *cis* (B) and *trans* (C) infection (*n* = 5). (D) SN MΦ were treated with a MAb against CD209 (DC-SIGN) or an isotype control and then assessed by flow cytometry for DC-SIGN detectability (*n* = 2). *, *P* ≤ 0.05. Histograms represent the means ± SE.

In addition to observed differences in the number of MΦ expressing DC-SIGN in NP compared to that in PR, the number of DC-SIGN^+^ MΦ positively correlated with *trans* infection efficiency in SN (Fig. 5A). To investigate further the role of DC-SIGN in HIV-1 *trans* infection, antibody blocking experiments were done as detailed in Materials and Methods. Blocking of DC-SIGN prior to virus exposure reduced MΦ susceptibility to *cis* infection by 72% compared to that of isotype-treated MΦ by day 12 (Fig. 5B). In addition, blocking DC-SIGN on MΦ prior to virus exposure and coculture with autologous CD4^+^ T cells reduced MΦ *trans* infection by 87% by day 12 (Fig. 5C). Blocked MΦ stained negative for DC-SIGN by flow cytometry, unlike isotype-treated MΦ, supporting a successful occlusion of DC-SIGN (Fig. 5D). These data indicate that a DC-SIGN-dependent mechanism of MΦ-virus engagement is pivotal for both MΦ *cis* and *trans* infection.

### NP MΦ have lower total cellular and cell membrane-associated free cholesterol than PR.

**FIG 6:**
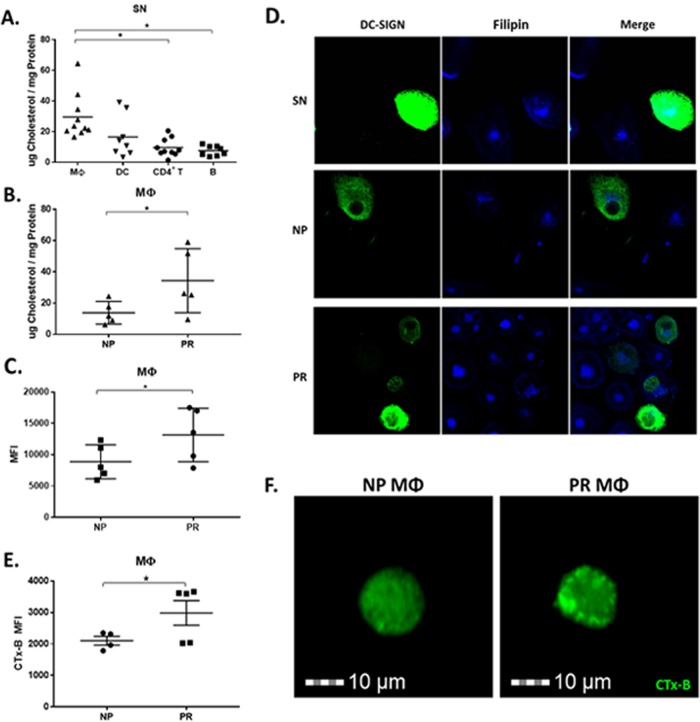
MΦ from NP have lower total cellular and cell membrane-associated free cholesterol than those from PR. (A) Total cholesterol measured by Amplex Red of SN MΦ (*n* = 10), DC (*n* = 8), activated CD4^+^ T cells (*n* = 10), and activated B cells (*n* = 8). (B) MΦ total cholesterol content was measured in NP and PR by Amplex red. (C) NP and PR MΦ were exposed to filipin for cell membrane cholesterol-specific labeling and analyzed by flow cytometry. (D) Representative images from SN, NP, and PR MΦ, labeled with filipin (blue) and DC-SIGN (green), by confocal microscopy. (E) MΦ were exposed to CTx-B for lipid raft labeling and analyzed by flow cytometry with a Millipore ImageStream. (F) Representative images of NP and PR MΦ labeled with CTx-B analyzed with a Millipore ImageStream. *, *P* ≤ 0.05. Histograms represent the means ± SE.

We have previously shown that alterations in cellular cholesterol metabolism correlate with the inability of DC and B lymphocytes from NP to efficiently *trans* infect CD4^+^ T cells (10). Therefore, we sought to determine if cellular cholesterol levels in MΦ were linked to HIV-1 *trans* infection and disease progression by quantifying total cholesterol, including esterified and unesterified free cholesterol (FC). In SN participants, total cholesterol was highest in MΦ, which was significantly higher than that in the B and T cells but not significantly different from that of DC. However, total cholesterol in DC trended lower than that in MΦ (Fig. 6A). When comparing the two HIV-1-infected groups, total cholesterol concentration of MΦ was significantly lower in NP than PR (Fig. 6B), suggesting that there is a cholesterol-dependent mechanism of *trans* infection in MΦ, in line with our previous findings in DC and B cells (10).

To further elucidate the effect of decreased MΦ cholesterol in NP, we assessed the level of cell membrane-associated FC as well as lipid rafting in NP and PR MΦ by flow cytometry using filipin III, a naturally occurring fluorescent polyene antibiotic that binds FC (23), and the lipid raft containing GM1-specific marker cholera toxin subunit B (CTx-B). Membrane-associated FC was significantly lower in NP MΦ than in PR (Fig. 6C). This characteristic was also reflected in confocal imaging analysis of filipin III of SN, NP, and PR MΦ. Both cholesterol and DC-SIGN staining appeared dimmer in NP than in SN and PR (Fig. 6D). Some PR MΦ had very high levels of lipid raft staining, and overall the lipid rafting detected in PR MΦ was significantly higher than that of NP (Fig. 6E). Furthermore, single-cell fluorescent images (acquired with the Millipore ImageStream X Mark II flow cytometer) revealed more punctate rafting of the CTx-B staining of PR compared to that of NP MΦ (Fig. 6F). This suggests that lipid raft distribution is different in NP than PR. Together, these data support that the presence of insufficient cholesterol in MΦ, specifically FC in MΦ cell membranes, could lead to the lack of MΦ-mediated *trans* infection in NP.

### SIMV decreases MΦ *trans* infection.

**FIG 7:**
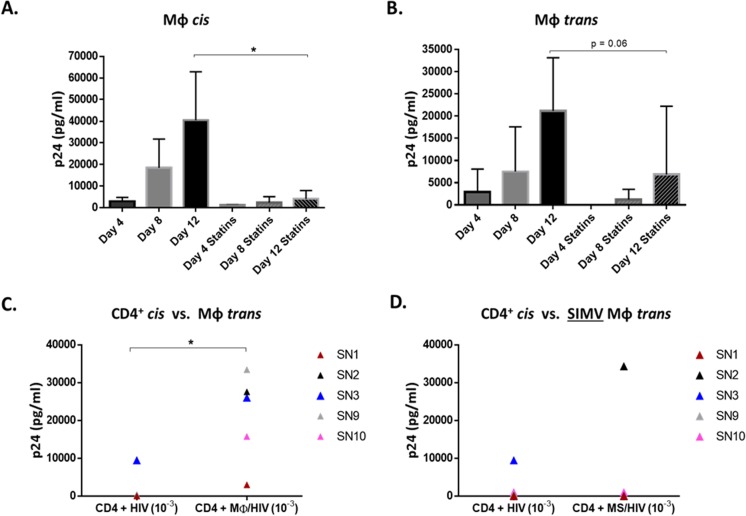
Statins lower MΦ *cis* and *trans* infection. (A and B) SN MΦ left untreated or treated with 1 μg/ml SIMV for 24 h were assessed for *cis* (A) and *trans* (B) infection (*n* = 4). Untreated (C) and SIMV-treated (D) MΦ (MS) *trans* infection efficiency was compared to autologous CD4^+^ *cis* infection at day 12. *, *P* ≤ 0.05. Histograms represent the means ± SE.

To test the role of cholesterol in *trans* infection, we assessed whether SIMV, an inhibitor of cholesterol synthesis, interfered with MΦ *cis* or *trans* infection. For this, MΦ treated with SIMV prior to virus exposure were used in *cis* and *trans* infection assays, as described in Materials and Methods. *cis* infection of MΦ was significantly decreased by 90% (Fig. 7A), and MΦ-mediated *trans* infection trended down sharply (*P* value of 0.06) (Fig. 7B) in the presence of SIMV. We then assessed the impact of SIMV on the ability of MΦ to enhance infection of CD4^+^ T cells in *trans* compared to CD4^+^ T cell infection with cell-free virus in *cis*. Consistent with the data shown in Fig. 1D, untreated MΦ loaded with HIV-1 significantly enhanced infection in *trans* compared to CD4^+^ T cell in *cis* infection by day 12 (Fig. 7C). However, SIMV-treated MΦ were unable to enhance infection in *trans* compared to *cis* infection in four of five SN samples tested (Fig. 7D). Although SIMV did not completely abrogate MΦ *cis* or *trans* infection, in both cases SIMV treatment led to a decrease in p24 production. Most importantly, SIMV blocked the ability of MΦ to significantly enhance *trans* infection of CD4^+^ T cells compared to *cis* infection.

### SIMV decreases DC-SIGN-expressing MΦ and *trans* infection in a cholesterol-mediated manner.

**FIG 8:**
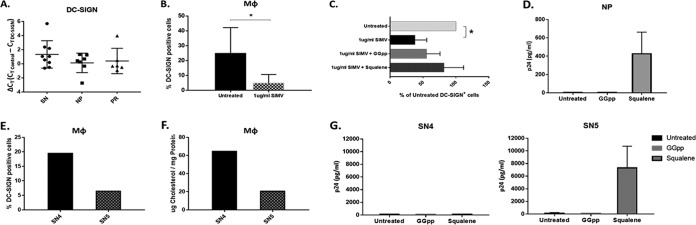
SIMV decreases the number of DC-SIGN^+^ MΦ and *trans* infection in a cholesterol-dependent manner. (A) Total RNA extracted from SN, NP, and PR MΦ was used to measure DC-SIGN gene expression by RT-PCR. The cycle threshold (*C_T_*) of the DC-SIGN probe was subtracted from the *C_T_* of the RNA polymerase II probe within each sample for a relative Δ*C_T_* value. (B) SN MΦ were left untreated or were treated with 1 μg/ml SIMV (*n* = 5) or in the presence of SIMV and GGpp or SIMV and squalene (C) for 24 h prior to analysis of DC-SIGN surface expression by flow cytometry (*n* = 4). (D) p24 level on day 12 of *trans* infection with untreated NP MΦ, NP MΦ treated with GGpp for 24 h, or NP MΦ treated with squalene for 24 h (*n* = 3). (E and F) Percentage of DC-SIGN^+^ MΦ (E) and total cholesterol (F) of participants SN4 and SN5. (G) p24 level on day 12 of *trans* infection with untreated SN4 and SN5 MΦ, SN4 and SN5 MΦ treated with GGpp for 24 h, or SN4 and SN5 MΦ treated with squalene for 24 h (two independent experiments). *, *P* ≤ 0.05. Histograms represent the means ± SE.

DC-SIGN RNA levels determined by reverse transcription-PCR (RT-PCR) were not different among SN, NP, and PR (Fig. 8A). We sought to determine if SIMV treatment decreased *trans* infection by altering DC-SIGN expression on the MΦ surface. SN MΦ treatment with SIMV followed by DC-SIGN analysis by flow cytometry revealed that SIMV decreased the number of DC-SIGN^+^ MΦ compared to that for untreated cells (Fig. 8B). SIMV is a known competitor of HMG-coenzyme A (CoA) reductase, and in addition to mediating cell cholesterol synthesis, HMG-CoA reductase also mediates protein prenylation in cells (24). Statins are known to interfere with this pathway in addition to cholesterol synthesis (25). Therefore, to elucidate by which pathway SIMV was altering DC-SIGN expression, SIMV-treated MΦ were simultaneously treated with either squalene or geranylgeranyl pyrophosphate (GGpp), downstream products of the cholesterol synthesis and protein prenylation pathways, respectively. In the presence of either squalene or GGpp, SIMV was unable to significantly decrease DC-SIGN, and the number of DC-SIGN^+^ MΦ trended higher with the squalene treatment than with GGpp (Fig. 8C). Additionally, squalene treatment, but not GGpp treatment, of MΦ recovered detectable *trans* infection in NP (Fig. 8D). This supports that low cholesterol in NP MΦ contributes to their inability to *trans* infect HIV-1.

Due to the lack of *trans* infection observed by SN4 and SN5 (Fig. 1D, E, and F), to further assess their similarity to NP, we compared their DC-SIGN expression and cholesterol levels. SN5 MΦ had lower levels of both DC-SIGN (Fig. 8E) and total cholesterol than SN4 (Fig. 8F). Although SN5 had lower total cholesterol, similar to NP, SN4 had cholesterol levels comparable to that of the *trans* infection-positive SN. Therefore, we tested the ability of both cholesterol synthesis and protein prenylation induction with squalene and GGpp, respectively, to recover *trans* infection in SN4 and SN5. Squalene, but not GGpp, recovered MΦ-mediated *trans* infection in SN5 but not SN4 (Fig. 8G). Thus, SN5 was both functionally and phenotypically similar to the NP group, while phenotyping suggests that the lack of *trans* infection by SN4 was caused by a mechanism independent of both cholesterol metabolism and protein prenylation.

### SIMV alters membrane-associated cholesterol and lipid rafting in MΦ.

**FIG 9:**
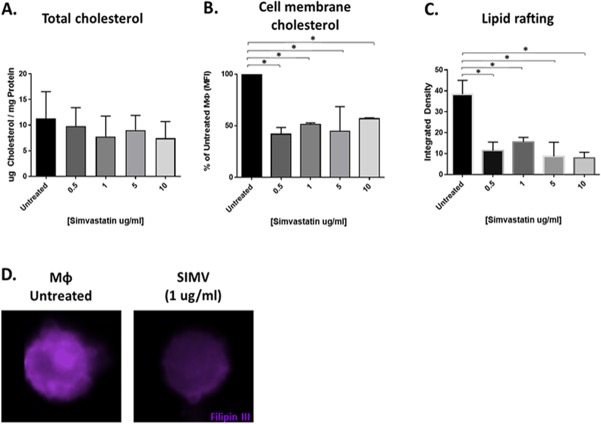
SIMV alters MΦ lipid rafting. (A and B) SN MΦ were treated with 0.5, 1, 5, or 10 μg/ml of SIMV and tested by Amplex red for total cholesterol concentration (*n* = 2) (A) and by flow cytometry for cell membrane cholesterol-specific staining with filipin (*n* = 2) (B). (C) MΦ were treated with 0.5, 1, 5, or 10 μg/ml of SIMV, exposed to CTx-B, and analyzed by confocal microscopy to quantify lipid rafting (*n* = 2). (D) Representative images from SN MΦ left untreated or treated with 1 μg/ml SIMV, followed by filipin labeling (purple) and imaging with the Millipore ImageStream. *, *P* ≤ 0.05. Histograms represent the means ± SE.

To further understand how SIMV alters MΦ cholesterol content and the role of cholesterol in MΦ-mediated *trans* infection, we analyzed the abundance of total cellular cholesterol compared to that of membrane-associated FC in SIMV-treated MΦ. Total cholesterol content of SIMV-treated MΦ did not decrease across a wide range of SIMV concentrations tested (0.5 to 10 μg/ml) (Fig. 9A). However, cell membrane-associated FC was lowered by SIMV (Fig. 9B), as demonstrated by analysis of cells treated with filipin III. Fluorescent CTx-B labeling of untreated and SIMV-treated MΦ showed that SIMV also significantly reduced plasma membrane lipid rafting (Fig. 9C). Intriguingly, visualization of filipin III cholesterol staining revealed a dissociation of FC clustering or rafting after 1 μg/ml SIMV treatment (concentration used in functional studies depicted in Fig. 7 and 8) and confirmed that cholesterol-rich lipid rafting was also lowered in SIMV-treated MΦ (Fig. 9D). These data suggest that SIMV effectively mimics the altered cholesterol state of MΦ observed in NP, the mechanism by which we propose SIMV significantly decreases HIV-1-mediated *trans* infection.

### PR MΦ express higher levels of PPARγ mRNA.

**FIG 10:**
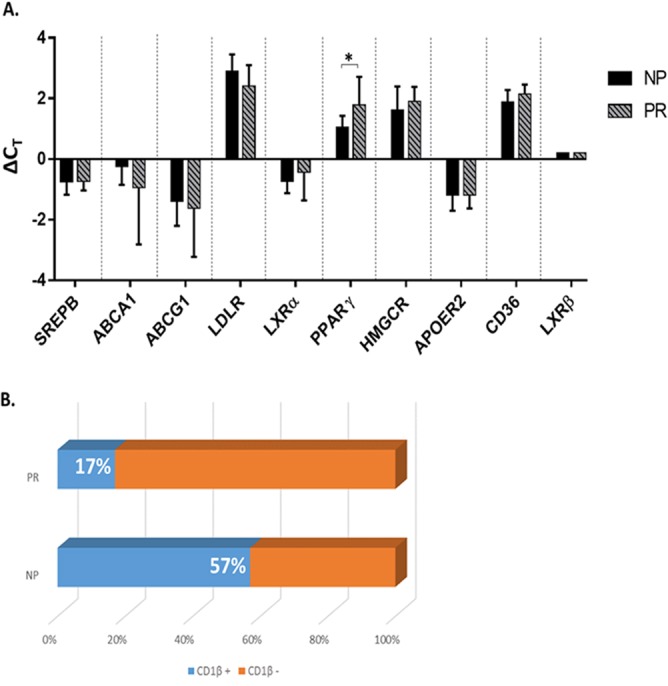
PR MΦ express higher levels of PPARγ mRNA. (A) Total RNA extracted from NP and PR MΦ was used to measure SREBP, ABCA1, ABCG1, LDLR, LXRα, PPARγ, HMGCR, APOER2, CD36, and LXRβ mRNA by RT-PCR. Histograms represent the means of technical triplicates ± SE. (B) CD1β mRNA levels were assessed by RT-PCR and are expressed as the number of samples with CD1β-expressing MΦ. *, *P* ≤ 0.05. Histograms represent the means ± SE.

We next sought to identify the mechanism by which levels of FC were being regulated differentially in NP and PR. Based on their well-defined roles in lipid sensing, uptake, efflux, and synthesis (26–29), the following proteins were selected for mRNA expression level analysis: sterol regulatory element-binding protein (SREBP), ATP-binding cassette transporter 1 (ABCA1), ATP binding cassette subfamily G member 1 (ABCG1), low-density lipoprotein receptor (LDLR), liver X receptor alpha (LXRα), peroxisome proliferator-activated receptor gamma (PPARγ), 3-hydroxy-3-methylglutaryl-CoA reductase (HMGCR), apolipoprotein E receptor 2 (APOER2), CD36, liver X receptor beta (LXRβ), and CD1β were generated from total RNA isolated from NP and PR MΦ. Preamplified cDNA was tested for expression levels by RT-PCR. We found that PPARγ expression was significantly higher in PR than NP, and that LDLR expression trended higher in NP (Fig. 10A). However, we found that ABCA1 expression was not significantly different in NP and PR (Fig. 10A), which is consistent with B cells and DC from NP and PR (10). Additionally, expression of SREBP, ABCG1, LXRα, HMCGR, APOER2, CD36, and LXRβ were not significantly different among NP and PR (Fig. 10A). CD1β was not expressed in all the MΦ samples but was in significantly more NP (4 of 7; 57%) than both PR (1 of 6; 17%) and SN (2 of 9; 22%) (Fig. 10B).

### NP MΦ internalize less HIV-1 into early endosomes.

**FIG 11:**
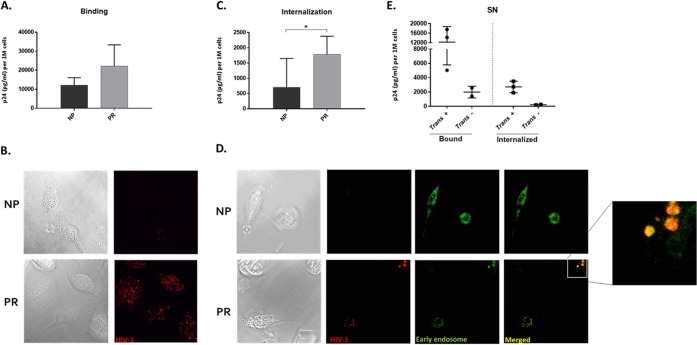
NP MΦ internalize less HIV-1 into early endosomes. (A) NP and PR MΦ were exposed to 12 ng p24 of AT-2-inactivated, CCR5-tropic HIV-1 per 0.5 × 10^6^ cells for 2 h at 4°C, lysed, and analyzed for bound p24 by ELISA (*n* = 4). (B) Representative differential interference contrast (DIC) and fluorescent images from SN MΦ treated with RFP-tagged, AT-2-inactivated CCR5-tropic virus (red) for binding assays analyzed by confocal microscopy. (C) NP and PR MΦ were exposed to 12 ng p24 of AT-2-inactivated CCR5-tropic HIV-1 per 0.5 × 10^6^ cells for 2 h at 37°C, trypsinized, lysed, and analyzed for internalized p24 by ELISA (*n* = 5). (D) Representative differential interference contrast (DIC) and fluorescent images from SN MΦ treated with RFP-tagged, AT-2-inactivated, CCR5-tropic virus (red) for virus internalization assays, costained for early endosomes (green), and analyzed by confocal microscopy. (E) MΦ from *trans* infection-positive and *trans* infection-negative SN were assessed for virus binding and internalization by p24 ELISA. *, *P* ≤ 0.05. Histograms represent the means ± SE.

Our data thus far indicated that DC-SIGN was playing a role in HIV-1 interactions with macrophages. To further understand the role of virion-MΦ interactions in HIV-1 disease progression, we assessed HIV-1 binding to and internalization into NP and PR MΦ. CCR5-tropic HIV-1 binding trended lower in NP MΦ than PR MΦ but was not significantly different (Fig. 11A). Confocal microscopy visually confirmed less virus binding in NP (Fig. 11B). DC-SIGN is known to mediate endocytosis of intact HIV-1 virions into early endosomes (16). We found that significantly less HIV-1 was internalized into NP MΦ (Fig. 11C). Additionally, very little internalized virus was visible in NP by confocal microscopy; however, internalized virus was more abundant in PR and colocalized with early endosomes (Fig. 11D). Once we determined the associations of less virus binding and internalization into NP MΦ, we assessed whether binding and internalization were lower in the 2 SN that lacked MΦ *trans* infection. We found that both binding and internalization of virus to and into MΦ was lower than that of SN, with efficient *trans* infection (Fig. 11E). Together these data support that virus-MΦ interactions, specifically virus internalization into early endosomes, play a role in MΦ-mediated *trans* infection and, thereby, disease progression.

## DISCUSSION

Here, we show that MΦ HIV-1 *trans* infection is deficient in NP and thus associated with lack of disease progression. As we have found in B cells and DC (10), the mechanism of poor MΦ *trans* infection of autologous CD4^+^ T cells is a result of altered cell cholesterol homeostasis. We further demonstrate that NP MΦ have significantly less unesterified FC and plasma membrane lipid rafting, which leads to fewer DC-SIGN-expressing MΦ and lower virus internalization into MΦ, resulting in a lack of *trans* infection.

We found that NP MΦ have significantly lower FC and membrane lipid rafting than PR MΦ. This corresponds with our previous finding that poor DC and B cell HIV-1 *trans* infection in NP is linked to altered cholesterol homeostasis (10). If not esterified and effluxed, FC in the cytosol is rapidly transported to the plasma membrane (PM), where it contributes to lipid raft formation which influences membrane fluidity and protein function (30–32). Blocking cholesterol synthesis in MΦ with SIMV significantly decreased FC, lipid rafting, and *trans* infection. *trans* infection was recovered in NP with squalene rather than GGpp treatment, suggesting cholesterol rather than protein prenylation is influencing *trans* infection ability.

NP and PR MΦ appear capable of sensing and responding to their cholesterol levels, indicated by their increased transcription of LDLR and PPARγ and leading to increased virus uptake or efflux, respectively (33). Additionally, more NP have CD1β-expressing MΦ, which better equips these cells for lipid antigen presentation and may ultimately associate with better anti-HIV-1 immune responses, a known characteristic of NP (34). However, based on our studies, the expression levels of many other genes involved in cholesterol uptake, efflux, and synthesis do not explain the mechanisms contributing to the differences in cholesterol levels we see in NP and PR MΦ, including ABCA1, which is consistent with our previous work with B cells and DC (10). This suggests that if greater cholesterol efflux is responsible for lower cholesterol in NP APC, it is mediated by an ABCA1-independent mechanism. Exosomes disseminated from HIV-1-infected cells have been shown to facilitate HIV-1 *trans* infection (35). Additionally, exosomes are known to transport proteins as well as RNAs, including microRNA (miRNA) (36). We hypothesize that the low-cholesterol state of NP APC is a result of extracellular factors, such as miRNA, signaling molecules, or apolipoproteins, all of which alter cellular cholesterol metabolism (37–39).

Our data show that SIMV treatment lowers the number of DC-SIGN-expressing MΦ from SN and their *trans* infection efficiency, and DC-SIGN blocking significantly decreases MΦ *trans* infection. Furthermore, fewer DC-SIGN-expressing MΦ are detected in NP compared to PR. DC-SIGN is constitutively expressed on MΦ in lymph nodes (40), as well as adult lung and uterine tissue (41). The C-type lectin also facilitates *trans* infection of HIV-1 from DC (18, 22), as well as B cells (19), to CD4^+^ T cells. It localizes in cholesterol-rich lipid rafts on the cell surface (42) and relies on rafting for efficient internalization of ligands (42, 43). Taken together, our data show that low FC content of NP MΦ membranes hinders lipid raft formation and thus functional surface expression of DC-SIGN, which, in turn, decreases efficiency of *trans* infection.

Importantly, SIMV treatment and DC-SIGN blocking did not completely abrogate MΦ *trans* infection. While DC-SIGN facilitated HIV-1 *trans* infection in our study, it is not the only mechanism by which MΦ mediate *trans* infection. HIV-1 can bind DC in a cholesterol-dependent manner, independent of CD4 and DC-SIGN (44). CD169 (Siglec-1) facilitates the capture of retroviruses by macrophages lining the sinus region of lymph nodes and its passage to B cells within follicles (45), as well as HIV-1 *trans* infection by DC (20). However, MΦ CD169 expression was not associated with *trans* infection in our studies. Analyses of MΦ isolated from lymphoid tissue from SN, PR, and NP will help to better understand the role of CD169 expression in HIV *trans* infection and its relationship to disease progression.

We have previously identified lack of APC-mediated *trans* infection as an inherent characteristic of NP, in that *trans* infection ability is similar pre- and post-HIV-1 seroconversion (10). Two of the 10 SN (SN4 and SN5) in our study lacked *trans* infection by all three types of APC. Both SN4 and SN5 exhibited lower HIV-1 internalization than *trans* infection-efficient SN. However, while SN5 MΦ had low cholesterol levels and DC-SIGN expression similar to that of NP, SN4 displayed no further NP characteristics and likely has a cholesterol-independent blockade in the *trans* infection pathway. This supports that the SN population is heterogeneous and comprised of individuals with genetically determined NP characteristics. Therefore, SN lacking *trans* infection ability represent a unique group of individuals from which novel factors of HIV-1 disease progression control can be identified.

*trans* infection occurs by endocytosis of intact virions, which can then be passed between cells through intercellular connections (46) or regurgitated into a synapse between two immune cells or by the budding of new virions as a result of productive *cis* infection into a similar synapse (9). The endocytosis pathway of *trans* infection is likely to be more heavily implicated in HIV-1 disease progression as the pathway shared by all three APC. We show that while NP and PR binding of HIV-1 is not different, NP MΦ internalize significantly less HIV-1 into early endosomes. We propose that decreased FC and lipid rafting in MΦ results in less DC-SIGN-facilitated endocytosis, thereby decreasing HIV-1 *trans* infection.

As has been previously demonstrated (47), we observed that SN and PR MΦ are significantly more susceptible to *cis* infection than NP MΦ. However, in SN, MΦ *cis* infection did not correlate with *trans* infection efficiency, as 5 of 10 SN with efficient MΦ *trans* infection had undetectable MΦ *cis* infection. Therefore, MΦ susceptibility to *cis* infection is not required for *trans* infection. Inefficient MΦ *cis* infection is likely not the sole factor responsible for the complete lack of *trans* infection observed in NP.

Changes in plasma membrane fluidity due to altered cholesterol and sphingolipid compositions can alter the expression and function of CD4 and CCR5 and HIV-1 *cis* infection (48–50). Although we did not see a difference in CD4 or CCR5 surface expression by NP and PR MΦ, membrane fluidity alterations could decrease the function of CD4, CCR5, and DC-SIGN, thereby impacting *cis* infection. We postulate that DC-SIGN enhances *cis* infection of MΦ, if not by directly facilitating viral envelope binding and fusion then by increasing or stabilizing virus interactions with the cell through gp120 interactions (51). MΦ in peripheral tissue, secondary lymphoid tissue, and the central nervous system harbor HIV-1 (52), and MΦ *cis* infection can occur in the presence of ART (53). DC-SIGN-facilitated *trans* infection mediated by the MΦ reservoir in PR would further explain persistence and maintenance of the T cell reservoir in such individuals despite ART. This mechanism of *trans* and *cis* infection could be hijacked by other viruses to enhance viral dissemination, as DC-SIGN (54, 55) and cholesterol are important for infection of many viruses (56–58).

In our studies, SIMV significantly decreased levels of FC, lipid rafting, and DC-SIGN-expressing MΦ and the ability of MΦ to enhance T cell infection in *trans* compared to that in *cis*. Collectively, studies suggest that the use of statins does not augment current HIV-1 treatment regimens (59–64); however, ART drugs can alter statin pharmacokinetics (65). Under alternative administration routes less affected by ART drugs, statins could reduce MΦ dissemination of HIV-1 and should be reconsidered for prophylaxis and therapy regimens. Manipulation of ABCA1- or ABCG-1-mediated cholesterol efflux using nuclear receptor agonists may serve a similar purpose.

**FIG 12:**
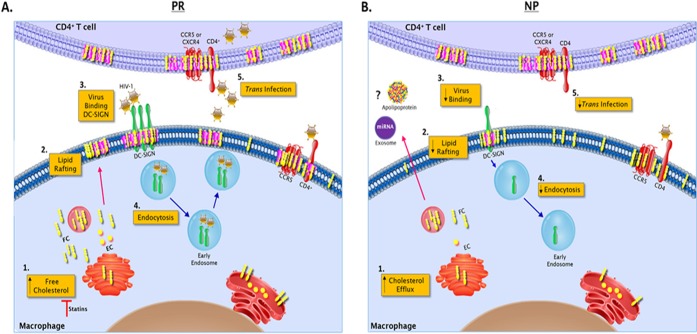
Schematic of the role of cholesterol in HIV-1 *trans* infection. (A) In HIV-1^+^ progressors, unesterified free cholesterol (FC) and esterified cholesterol (EC) are abundant in the MΦ and T cell (1), specifically in the plasma membrane, where it aggregates to form lipid rafts (2). (3) Abundant lipid rafting allows for abundant DC-SIGN expression on the MΦ surface and HIV-1 binding. (4) This leads to DC-SIGN-mediated virion endocytosis, intracellular transport, and release of virus into the virological synapse, where optimal infection of CD4^+^ T cells occurs in *trans*. (5) At low MOIs, *trans* infection occurs in the absence of MΦ and T cell *cis* infection. (B) In HIV-1^+^ nonprogressors, MΦ display significantly lower levels of total cholesterol (1), which we hypothesize is mediated by extracellular elements such as miRNA, signaling molecules, or apolipoproteins facilitating greater cholesterol efflux. (2) Subsequently, lower levels of FC in the plasma membrane results in significantly less lipid rafting and DC-SIGN expression. Consequently, DC-SIGN-mediated virus binding (3) and virion endocytosis (4) do not occur at a rate sufficient enough to mediate CD4^+^ T cell infection in *trans* (5).

In sum, we have shown that DC-SIGN-facilitated MΦ *trans* infection is associated with HIV-1 disease progression. We demonstrate the importance of FC-rich cells and lipid rafting for DC-SIGN-mediated endocytosis of HIV-1 and subsequent *trans* infection of CD4^+^ T cells observed in PR that is lacking in NP (Fig. 12). Additionally, SIMV treatment lowered the DC-SIGN^+^ MΦ population, lipid rafting, and MΦ *cis* and MΦ *trans* infection efficiency, all features characteristic of NP. Consequently, lowering APC cholesterol in combination with antivirals should be considered in treating HIV-1 infection. By targeting *trans* infection in addition to *cis* infection, we could further reduce dissemination of virus and the size of the latent reservoir.

## MATERIALS AND METHODS

### Ethics statement.

All recruited participants were over the age of 18 years and provided written consent prior to biologic sample collection or use according to University of Pittsburgh Internal Review Board-approved protocols.

### Participants.

We studied three groups of individuals (Table 1) within the Pittsburgh clinical site of the MACS (Pitt Men's Study) defined based on their HIV-1 serostatus and associated disease progression: (i) for PR, HIV-1 seropositive individuals with a 100-cells/mm^3^ annual decrease in CD4^+^ T cell count prior to ART; (ii) for NP, HIV-1-seropositive individuals displaying a lack of progression to AIDS and CD4^+^ T cell counts above 500 for at least 7 years postseroconversion without the aid of ART; and (iii) for SN, healthy HIV-1-negative individuals whose HIV-1 serostatus has been checked biannually. We recruited 10 SN as well as 5 PR and 5 NP not on ART at the time of study with cryopreserved samples available prior to ART initiation. Participant-reported data ruled out the use of cholesterol- or lipid-lowering medications during the time the blood samples were obtained. HLA B locus genotype and CCR5 Δ32 genotype were considered for all participants as possible confounders of disease progression classification.

### Cell isolation and culture.

CD4^+^ T lymphocytes, B lymphocytes, and monocytes were positively enriched from freshly isolated or frozen PBMC using anti-CD4, -CD19, or -CD14 monoclonal antibody (MAb)-coated magnetic bead separation (Miltenyi Biotech) according to the manufacturer's instructions. MΦ and DC were derived from monocytes cultured with 20 ng/ml macrophage colony-stimulating factor (M-CSF; Peprotech) for 7 days or 1,000 U/ml granulocyte-macrophage colony-stimulating factor (GM-CSF; Miltenyi Biotech) and 1,000 U/ml recombinant human interleukin-4 (rhIL-4; R&D Systems) for 5 day in AIM-V medium (Gibco). CD4^+^ T cells and B cells were activated for 48 h with 10 U/ml of delectinated IL-2 and 2 μg/ml phytohemagglutinin (PHA) (9) or 1,000 U/ml rhIL-4 and 0.1 μg/ml CD40L, respectively.

R5-tropic HIV-1 BaL, grown in and purified from PM1 cells (American Type Culture Collection) (66), was used for *cis* and *trans* infection experiments. Virus stock titration and experimental p24 measurements were acquired by enzyme-linked immunosorbent assay (ELISA) using the HIV-1 p24 antigen capture immunoassay kit (Leidos Biomedical Research Inc., Frederick National Laboratory for Cancer Research) per the manufacturer's instructions.

### *trans* and *cis* infections. (i) *trans* infection.

A total of 1 × 10^6^ APC were incubated with a low concentration of HIV-1 (MOI, 10^−3^) for 2 h at 37°C and then washed 3 times with medium. Virus-loaded APC were cocultured with autologous activated CD4^+^ T cell targets at a 1:10 effector/target ratio (10^4^:10^5^) in AIM-V medium, and p24 was quantified in cell-free supernatants at days 4, 8, and 12 postcoculture.

### (ii) *cis* infection.

A total of 1 × 10^6^ APC or activated CD4^+^ T cells were incubated with low (10^−3^) or high (10^−1^) MOIs of HIV-1 and cultured independently. The p24 levels were measured in cell-free supernatants at days 4, 8, and 12 postcoculture.

### Cell phenotyping.

Cells were assessed for surface and intracellular protein expression. MΦ were incubated with an amine-binding viability dye using the LIVE/DEAD fixable aqua dead cell stain kit (Molecular Probes) for 20 min and then incubated with isotype controls or monoclonal antibodies against CD4 (V450), CD14 (APC-H7), CD16 (APC-H7), CCR5 (phycoerythrin), CD169 (BB515) (BD Biosciences), and CD209 (APC or fluorescein isothiocyanate [FITC]) (R&D Systems) diluted in wash buffer (phosphate-buffered saline [PBS] supplemented with 0.1% fetal calf serum and 0.1% NaN_3_) for 30 min. Cells were then permeabilized with Perm II (BD Biosciences) for 20 min and labeled intracellularly with anti-CD68 (BV421; BD Biosciences) for 30 min. Cells were fixed in 1% paraformaldehyde (PFA), acquired with a BD LSR Fortessa, and analyzed with FlowJo v10. Fluorochrome compensation was performed using an anti-mouse Ig, κ/negative-control compensation particles set (BD Biosciences) and ArC amine-reactive compensation bead set (Thermo Fisher) prior to acquisition.

### Cellular cholesterol quantification. (i) Total cellular cholesterol.

Cultured cells were washed two times with PBS to remove any residual media and lysed with 200 μl of 0.1% Triton X-100 for total cellular cholesterol quantification using the Amplex red cholesterol assay kit the per manufacturer's instructions (Thermo Fisher). Total cholesterol content was normalized to total cellular protein quantified with the micro-bicinchoninic acid (BCA) protein assay kit per the manufacturer's instructions (Thermo Fisher). Briefly, total cholesterol (in micrograms) was divided by total protein (in milligrams) for each sample.

### (ii) Membrane-associated cholesterol.

Cultured cells were analyzed for membrane-associated cholesterol using filipin III from the cholesterol cell-based detection assay kit (Cayman Chemical), per the manufacturer's instructions, and analyzed by flow cytometry or confocal microscopy. For flow-cytometric analysis, cells were incubated with viability dye, labeled with filipin III, resuspended in 1% PFA, acquired with a BD LSR Fortessa, and analyzed with FlowJo v10 or acquired with the EMD Millipore Amnis ImageStreamX and analyzed with IDEAS 6.2. For confocal microscopy analysis, MΦ were grown in glass-bottom dishes coated with poly–d-lysine (MatTek). Cells were fixed and labeled with filipin III reagent according to the manufacturer's instructions. Cells additionally stained for DC-SIGN with anti-CD209 (FITC; BD Biosciences) were noted. Cells were acquired on a Nikon A1 confocal microscope at 40× with a 3.42× zoom at the University of Pittsburgh Center for Biological Imaging and analyzed using NIS Elements.

### (iii) Plasma membrane lipid rafts.

In addition to cholesterol-specific labels, the GM1 ganglioside was labeled using the Vybrant Alexa Fluor 488 lipid raft labeling kit (Molecular Probes) and analyzed by flow cytometry or confocal imaging. Integrated density was calculated with ImageJ2 from an average of five cells per image using five images per sample.

### Cholesterol synthesis and protein prenylation studies. (i) Statin treatment.

MΦ were washed free of AIM-V media after 6 days of culture and then treated for 24 h with a range of concentrations (0.5 to 10 μg/ml) of simvastatin (SIMV) (Sigma) in RPMI supplemented with charcoal-stripped fetal bovine serum (FBS; Gibco) and M-CSF prior to downstream assays.

### (ii) Statin recovery.

During 24 h of incubation with SIMV, cells were simultaneously treated with squalene (Sigma) to recover cholesterol or geranylgeranyl pyrophosphate (GGpp) (Sigma) to recover protein prenylation blocked by statin treatment. Cells were then washed extensively prior to use in downstream assays.

### (iii) Cholesterol repletion.

After day 7 of culture, MΦ were incubated with 300 μg/ml of soluble cholesterol in BCD (Sigma) for 2 h and then washed prior to use in downstream assays.

### (iv) cDNA preamplification and RT-PCR.

Total RNA was extracted from MΦ with the RNeasy minikit (Qiagen), and cDNA was generated per the manufacturer's instructions. cDNA corresponding to human DC-SIGN, SREBP, ABCA1, ABCG1, PPARγ, LXRα, LXRβ, LDLR, CD36, HMG-CoA reductase, APOE receptor 2, and CD1β was preamplified using the TaqMan PreAmp master mix kit (Applied Biosystems) per the manufacturer's instructions. CD1β was also preamplified using IDT TaqMan primers (primer 1, 5′ACTTTTGGGCTGATATCTTGGG-3′; primer 2, 5′-CTTCCTTGCTCCTTTTGCTATG-3′; probe, 5′-/56-FAM/CTCATG GGA/ZEN/TCTGATATGACCGGCG/31ABkFQ/-3′). Preamplified cDNA was then used for RT-PCR using TaqMan gene expression assays (Applied Biosystems) (SREBP, Hs01088679_g1; ABCA1, Hs01059137_m1; ABCG1, HS00245154_m1; PPARγ, Hs01115513_m1; LXRα, Hs00172885_m1; LXRβ, Hs01027215_g1; LDLR, Hs00181192_m1; CD36^−^, Hs00354519_m1; HMGCR, Hs00168352_m1; and APOER, Hs00182998_m1) and TaqMan Universal master mix II, no UNG. RNA polymerase 2 (RNA pol2) was amplified from each sample as an internal control. Δ*C_T_* values were calculated by subtracting averaged threshold cycle (*C_T_*) values of the gene of interest from the RNA pol2 *C_T_* within each sample.

### Virus binding and internalization studies. (i) Binding.

MΦ were incubated with 12 ng of p24 per 0.5 × 10^6^ cells of AT-2 inactivated BaL HIV-1 for 2 h at 4°C, washed extensively with cold PBS, pelleted, and lysed with 0.1% Triton X-100. Cell lysates were assessed for p24 content by ELISA. For confocal analysis, cells were incubated with AT-2-inactivated red fluorescent protein (RFP)-tagged BaL HIV-1 for 2 h at 4°C, washed, mounted, and analyzed by confocal microscopy.

### (ii) Internalization.

MΦ were incubated with 12 ng of p24 per 0.5 × 10^6^ cells of AT-2-inactivated BaL HIV-1 for 2 h at 37°C, washed extensively with PBS, trypsinized to remove surface-bound virus, pelleted, and lysed with 0.1% Triton X-100. Cell lysates were assessed for p24 content by ELISA. For confocal analysis, CellLight Early Endosomes-GFP, BacMam 2.0 (Thermo Fisher), was added on day 6 of culture and incubated overnight. Cells were then washed and incubated with AT-2-inactivated RFP-tagged BaL HIV-1 for 2 h at 37°C, washed, mounted, and analyzed by confocal microscopy.
